# A Novel Sustained Anti-Inflammatory Effect of Atorvastatin—Calcium PLGA Nanoparticles: In Vitro Optimization and In Vivo Evaluation

**DOI:** 10.3390/pharmaceutics13101658

**Published:** 2021-10-11

**Authors:** Dalia H. Abdelkader, Ahmed Kh. Abosalha, Mohamed A. Khattab, Basmah N. Aldosari, Alanood S. Almurshedi

**Affiliations:** 1 Pharmaceutical Technology Department, Faculty of Pharmacy, Tanta University, Tanta 31111, Egypt; ahmed.khaled@pharm.tanta.edu.eg; 2 Department of Cytology and Histology, Faculty of Veterinary Medicine, Cairo University, Giza 12211, Egypt; mabdelrazik@cu.edu.eg; 3 Department of Pharmaceutics, College of Pharmacy, King Saud University, Riyadh 11451, Saudi Arabia; baldosari@ksu.edu.sa

**Keywords:** atorvastatin calcium, poly(lactide-co-glycolide), polymeric nanoparticles, carrageenan induced inflammation, anti-inflammatory, sustained release

## Abstract

Atorvastatin Calcium (At-Ca) has pleiotropic effect as anti-inflammatory drug beside its main antihyperlipidemic action. Our study was conducted to modulate the anti-inflammatory effect of At-Ca to be efficiently sustained for longer time. Single oil-water emulsion solvent evaporation technique was used to fabricate At-Ca into polymeric nanoparticles (NPs). In vitro optimization survey was performed on Poly(lactide-co-glycolide) (PLGA) loaded with At-Ca regrading to particle size, polydispersity index (PDI), zeta potential, percent entrapment efficiency (% EE), surface morphology and in vitro release pattern. In vitro drug-polymers interactions were fully scanned using Fourier-Transform Infrared Spectroscopy (FTIR) and Differential Scanning calorimetry (DSC) proving that the method of fabrication is an optimal strategy maintaining the drug structure with no interaction with polymeric matrix. The optimized formula with particle size (248.2 ± 15.13 nm), PDI (0.126 ± 0.048), zeta potential (−12.41 ± 4.80 mV), % EE (87.63 ± 3.21%), initial burst (39.78 ± 6.74%) and percent cumulative release (83.63 ± 3.71%) was orally administered in Male Sprague–Dawley rats to study the sustained anti-inflammatory effect of At-Ca PLGA NPs after carrageenan induced inflammation. In vivo results demonstrate that AT-Ca NPs has a sustained effect extending for approximately three days. Additionally, the histological examination revealed that the epidermal/dermal layers restore their typical normal cellular alignment with healthy architecture.

## 1. Introduction

Statins are classified according to their solubility into lipophilic such as atorvastatin, lovastatin and simvastatin or hydrophilic-like pravastatin and fluvastatin. In addition, they are divided into two generations depending on their originality: naturally occurring (simvastatin and pravastatin) or chemically synthetized (e.g., atorvastatin and rosuvastatin) agents. Generally, statins have various pharmacokinetics and pharmacodynamic properties affecting on their ADME (Absorption, distribution, Metabolism and Elimination) behavior, lipid reducing actions and side effects upon administration [1].

Basically, the antihyperlipidemic effect of statins includes inhibition of HMG-CoA (β-Hydroxy β-methylglutaryl-CoA) reductase enzyme which converts 3-hydroxy-3-methylglutaryl-CoA into L-mevalonate (the main precursor for cholesterol formation). L-mevalonate pathway has several biofunctions beside cholesterol production, e.g., enhancing cellular proliferation and migration, controlling formation of myelin sheath and dynamicity of filamentous network of cellular proteins. This ensures that statins have some auxiliary pharmacological effects known as “pleiotropic effects” [2].

**Figure 1:**
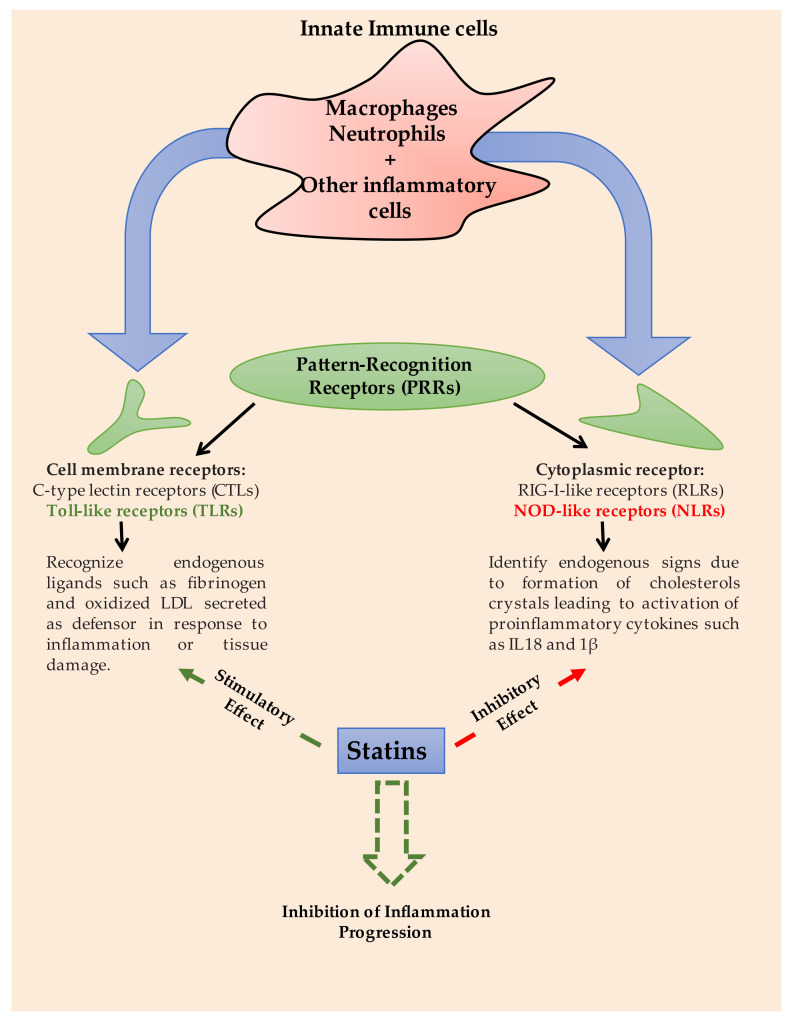
Pharmacological pathways of statins to inhibit inflammation progression via drug-receptors interactions of innate immune cells [1].

Recently, several studies investigated the anti-inflammatory action of statins independently on their lipid lowering effect [1,2,3,4]. The pattern recognition receptors (PRRs) of different immune cells such as macrophages, neutrophils, dendritic cells and etc. are located on cell membrane such as C-type lectin receptors (CTLs) and Toll-like receptors (TLRs) or embedded in the cytoplasmic microenvironments such as RIG-I-like receptors (RLRs) and NOD-like receptors (NLRs) [1]. Specially, NLRs and TLRs are two crucial types acting as a link to induce or inhibit the inflammation process as summarized in Figure 1. Statins can efficiently interrupt the inflammation cycle via inhibiting and stimulating the action of NLRs and TLRs, respectively [1]. Rosuvastatin and atorvastatin could suppress the activation of T cells through oxidized LDL (low-density lipoprotein). T cell activation via the influence of dendritic cells plays a major role in flaring up the inflammation [5]. Statins could disrupt the miRNA sequencing which effectively regulate NF-ĸB levels in dendritic cells leading to NF-ĸB deactivation as illustrated in Frostegård et al., study [5]. Other supposed anti-inflammatory mechanism is that statin could modulate cellular level of Rho proteins, one of small guanine triphosphate (GTP) binding proteins, which is highly expressed in pro-inflammatory cytokines [6].

Our model drug atorvastatin calcium (At-Ca) has various formulation obstacles preventing implementation of desired release pattern with high bioavailability [7]. At-Ca has poor aqueous solubility with low oral bioavailability approximately equal to 14% due to extensive first pass hepatic metabolism [8]. Different formulation strategies have been utilized to overcome these obstacles such as solid dispersion [9], co-crystallization [10], incorporation of self-emulsifying vehicle [11], etc.

Our approach is to fabricate At-Ca into polymeric PLGA nanoparticles (NPs). PLGA is a biodegradable and biocompatible polymer [12]. It is safely employed in oral delivery systems [13]. Encapsulation powered drug into nanosized polymeric matrix will enhance aqueous drug solubility. Drug particles would be amorphously formulated achieving higher dispersibility with better wetting properties [14]. NPs could efficiently escape enzymatic degradation occurring in liver and gastrointestinal tract. NPs reach directly to systemic circulation via lymphatic pathway through M-cells [7]. Polymeric NPs is ultimately considered the optimum delivery system for drug candidate sensitive to first pass metabolism with poor aqueous solubility.

At-Ca has been previously formulated into polymeric nanoparticles. Zhenbao Li et al., used probe ultrasonication and evaporation method to prepare At-Ca loaded PLGA NPs. The oral bioavailability of At-Ca has been enhanced to approximately 4 folds comparing with free At-Ca suspension [15]. Additionally, At-Ca chitosan nanocrystals fabricated into PLGA polymeric background showed minimum drug degradation, optimized pharmacokinetic parameters and efficient hypolipidemic action [16]. Furthermore, brain delivery of At-Ca loaded amphiphilic PLGA-b-PEG NPs was investigated by Soner Şimşek et al., demonstrating that the concentration of polyethylene glycol (PEG) and polysorbate 80 have a determining effect on the particle size, drug loading efficiency, release behavior concurrently with the cellular uptake to endothelial cells. They concluded that At-Ca loaded amphiphilic PLGA-b-PEG NPs, able to penetrate the blood brain barrier achieving significant targeting 1 h post injection, are considered promising nanocarriers for treatment of several neurological disorders [17]. In this manuscript, we are concerning with the anti-inflammatory effect of atorvastatin calcium (At-Ca) after carrageenan induced inflammation in Male Sprague–Dawley rats. We study the effect of encapsulating At-Ca into PLGA NPs and compare it with free At-Ca and placebo NPs (contain no medication).

## 2. Materials and Methods

### 2.1. Materials

Poly (D,L-lactide-co-glycolide, acid terminated, lactide:glycolide 50:50, MW 24,000–38,000), Poly (vinyl alcohol) (MW = 31,000–50,000, 87–89% hydrolysed), poly (ethylene glycol) (PEG) flakes (MW 2000 Da), Carrageenan, suitable for gel preparation, were all purchased from Sigma Aldrich, Gillingham, UK. Atorvastatin calcium was gifted from El Obour Modern Pharmaceutical Industries-Egypt. Cal-Ex™ II Fixative/Decalcifier reagent was purchased from Fisher Scientific, Pittsburgh, PA, USA). Phosphate Buffered Saline Tablets, Dulbecco A (PBS, Oxoid^TM^, ThermoFisher Scientific, Waltham, MA, USA). Dichloromethane (DCM), dimethylformamide (DMF) and sodium chloride (NaCL) were all purchased from Al—Gomhoria Co. for medicines and medical supplies, Egypt. All other used solvents, reagents and chemical are of appropriate standards with no further purification needed. Millipore^®^ deionized water was used throughout all the experiments.

### 2.2. Atorvastatin Calcium (At-Ca) PLGA NPs Fabrication

At-Ca loaded NPs were prepared using single oil-water emulsion solvent evaporation technique [18] with slight modifications for proper NPs fabrication. Firstly, PEG (5% *w*/*w*) comixed with different concentrations of PLGA (% *w*/*v*) were dissolved in DCM (Table 1). The weight ratio between PLGA and PEG was kept to being 19:1 in all NPs formulae (Table 1). At-Ca (10 mg) was dissolved in DMF. Then, the organic phase with volume ratio of DMF (1): DCM (9) was well vortexed for 2 min. O/W emulsion was formed by probe homogenization (Cole-Parmer Model 50 Cp T 4710 Series Ultrasonic Homogenizer, Chicago, IL, USA) at an operating frequency 20 KHz for 3 min of the organic phase into the aqueous phase containing diverse concentrations of PVA (% *w*/*v*) as shown by the formulae codes in Table 1. The organic/aqueous phase volume ratio was fixed at 1:100 throughout the study The prepared formulae were stayed overnight on magnetic stirring to allow the evaporation of organic solvents and ensure solidification of NPs. The nanoparticles were then collected by centrifugation (Hettich Microliter centrifuge MIKRO 220, Tuttlingen, Germany) at 10,000× *g* for 20–25 min at 4 °C and washed three times with deionized water to remove excess PVA and free unentrapped At-Ca in supernatants [7]. Three individually prepared batches of At-Ca PLGA NPs were employed for each data point’s analysis for in vitro characterizations mentioned in Section 2.3, Section 2.4 and Section 2.7. The mean ±Standard Deviation (SD) was calculated.

### 2.3. Particle Size and Zeta Potential Measurements

Particle size (diameter, nm), polydispersity index (PDI) and zeta potential of At-Ca PLGA NPs were determined using laser diffraction technique (NanoBrook 90Plus, Brookhaven Instruments Corporation, New York, NY, USA). The prepared At-Ca PLGA NPs samples were appropriately diluted to tenfold of its volume with deionized water for proper analysis. The measurement parameters were adjusted (wavelength (658 nm), viscosity (0.798 cP) and refractive index (1.33)) using Broakhaven software.

### 2.4. Determination of Percent Entrapment Efficiency (EE)

The amount of At-Ca encapsulated into polymeric matrix composed from PLGA and PEG (19:1) was determined indirectly by assaying the free unentrapped drug dispersed in the supernatant obtained after centrifugation step mentioned in Section 2.2 [19]. The amount of At-Ca was measured using UV-visible spectrophotometry (cuvette type) at detection wavelength of 282 nm (Evolution 300 spectrophotometer, Thermo Scientific, Waltham, MA, USA) [7]. The indirect % EE was calculated as follow:

(1) $$\mathrm{Indirect}\;\%\mathrm{EE}=\frac{\mathrm{Total}\;\mathrm{mass}\;\mathrm{of}\;\mathrm{drug}\;\mathrm{used}\;(\mathrm{mg})-\mathrm{mass}\;\mathrm{of}\;\mathrm{drug}\;\mathrm{in}\;\mathrm{supernatant}\;(\mathrm{mg})\;}{\;\mathrm{Total}\;\mathrm{mass}\;\mathrm{of}\;\mathrm{drug}\;\mathrm{used}\;(\mathrm{mg})\;}\times \;100$$

### 2.5. Morphological Imaging

At-Ca PLGA NPs were loaded on carbon coated Cu grids (200 mesh) and kept for appropriate time till fixation. At-Ca PLGA NPs were visualized using transmission electron microscope (TEM) at 200 kV (TEM, HRTEM, JEM 2100, JEOL, Tokyo, Japan).

### 2.6. Analysis of Drug—Polymer Interaction

#### 2.6.1. Fourier-Transform Infrared Spectroscopy (FTIR)

The FTIR spectrophotometer (Bruker Tensor 27, Borken, Germany) was employed for detecting the FTIR spectral bands of PLGA 50:50, PEG2000, pure At-Ca and the prepared At-Ca NPs (F5). The samples in solid state were physically mixed with potassium bromide and compressed in spherical disks. FTIR was run in the range of 4000 to 400 cm^−1^ for each sample.

#### 2.6.2. Differential Scanning Colorimetry (DSC)

Thermal scanning of PLGA 50:50, PEG 2000, free At-Ca and At-Ca NPs (F5) were investigated using differential scanning calorimeter (PerkinElmer, Inc. STA 6000, Waltham, MA, USA). Before starting, nitrogen gas should be purged at a rate equal to 40 mL/min through DSC cell. Heating rate was fixed at 10 °C/min to achieve equality of thermal scanning. the endothermic peaks were recorded in the range of 20–200 °C.

### 2.7. In Vitro Drug Release

In vitro release platform of different At-Ca PLGA NPs formulae was fully investigated using dialysis membrane method. Bags of dialysis membrane (Molecular weight cut off 12–14 kDa Fisher Scientific, Pittsburgh, PA, USA) were soaked overnight in release media (Phosphate Buffer Saline, PBS, 7.4) prior to in vitro release experiment. Using beaker 100 mL, the prepared nano-formulations containing At-Ca equivalent to 10 mg were placed in dialysis bags that should be tightly sealed to avoid leakage. Sink condition was maintained by adjusting the volume of release medium to be three times larger that is required to prepare a saturated solution of At-Ca [20]. Dialysis bag was immersed in 50 mL PBS and stirred at 37 °C. At predetermined time intervals (1, 2, 4, 6, 8, 24, 48 and 72 h), two mL samples were withdrawn for analysis. Same volume of fresh medium should be replaced to keep the volume constant at 50 mL [7]. The concentrations of At-Ca in collected samples were assayed using UV-visible spectroscopy as mentioned before in Section 2.4. A release of free At-Ca was performed as a control to evaluate the effect of PLGA NPs on the sustained release of nano-formulations. Each experiment was carried out in triplicate and the percent cumulative release was expressed as a mean ± SD.

### 2.8. In Vivo Study

All animal experiments including handling, study design and euthanization were carried out in accordance with the rules approved by Faculty of Pharmacy and the University of Tanta’s Animal Ethics Committee guidelines.

#### 2.8.1. Induction of Carrageenan Induced Inflammation

Carrageenan induced inflammation was initiated by subcutaneous (S.Q) injection of carrageenan solution freshly prepared in normal saline [21]. The experimental animal model was kept unanesthetized during S.Q injection of 0.2 mL carrageenan solution (1% *w*/*v*) in subplanter right hind paw. The left hind paw acts as control [22].

#### 2.8.2. Experimental Groups

Five groups (n = 6, at each time interval) of male Sprague–Dawley rats weighing 200–250 g were employed throughout this study. The first group serves as a standard non-inflamed model, not injected with carrageenan. Half an hour before carrageenan injection, normal saline (untreated control group), placebo NPs (contain no drug), free At-Ca and At-Ca PLGA NPs were orally administered to the other four remaining groups. All groups were fasted for approximately eight hours before receiving the medication. The amount/mass of AT-Ca PLGA NPs was calculated to adjust At-Ca equivalent to 10 mg/kg of rat’s weight [7]. At different three-time intervals of 6, 24 and 72 h, all right and lefts paws should be precisely excised at the same place and weighed within maximally 0.5 h to avoid water loss and hence weight variation [22]. The percent decrease in paw edema’s weight was calculated using this equation:

(2) $$\%\;\mathrm{Decrease}\;\mathrm{in}\;\mathrm{paw}\;\mathrm{edema}’s\;\mathrm{weight}=\frac{Wt\;left-Wt\;right}{Wt\;left}\;\times \;100$$

where, *Wt left* and *Wt right* are weights of left (control, not injected) and right (injected with carrageenan) paws, respectively. Only numerical results were considered regardless of the negative sign.

#### 2.8.3. Histological Examination

Paws samples were fixed in 10% neutral buffered formalin for 48 h followed by decalcification using Cal-X II for 14 days, then mid sagittal section was made to the whole paws using a surgical blade, followed by samples processing using serial grades of ethanol, cleared in xylene followed by infiltration and embedding in paraplast tissue embedding media. Five microns 2–3 step serial tissue sections were made by rotatory microtome from each sample and mounted on glass slides for hematoxylin and eosin (H and E) staining for general histological examination of tissue samples and grading of recorded cutaneous lesions. All guidelines for samples fixation, processing and staining were carried out according to Culling techniques [23]. Samples were examined and micrographs were obtained by Leica Full High Definition (HD) microscopic imaging system (Leica Microsystems GmbH, Wentzler, Germany).

### 2.9. Statistical Analysis

All experiments were performed for at least in triplicates. Results are shown as the mean ±SD. Prism 5 (Graph-Pad Software) was used for statistical analysis. A one-way ANOVA, followed by a pairwise comparison post-hoc test, were utilized wherever appropriate. (*p* < 0.05) is considered statistically significant.

## 3. Results

In this study, two formulation variables (PLGA and PVA concentrations) with three levels of each were conducted as demonstrated in Table 1. The influences of these two independent factors were screened on the mean particle size, PDI, zeta potential, entrapment efficiency and In vitro release pattern of At-Ca NPs. TEM was employed to examine NPs morphology and any interaction occurred between the polymeric content and the encapsulated drug was investigated using FTIR and DSC. In vivo study was further performed to compare the anti-inflammatory effect of placebo NPs (contain no drug), free At-Ca and the optimized At-Ca NPs that showed a well-controlled in vitro characterization.

### 3.1. Effect of Formulation Variables

#### 3.1.1. PLGA Concentration

**Figure 2:**
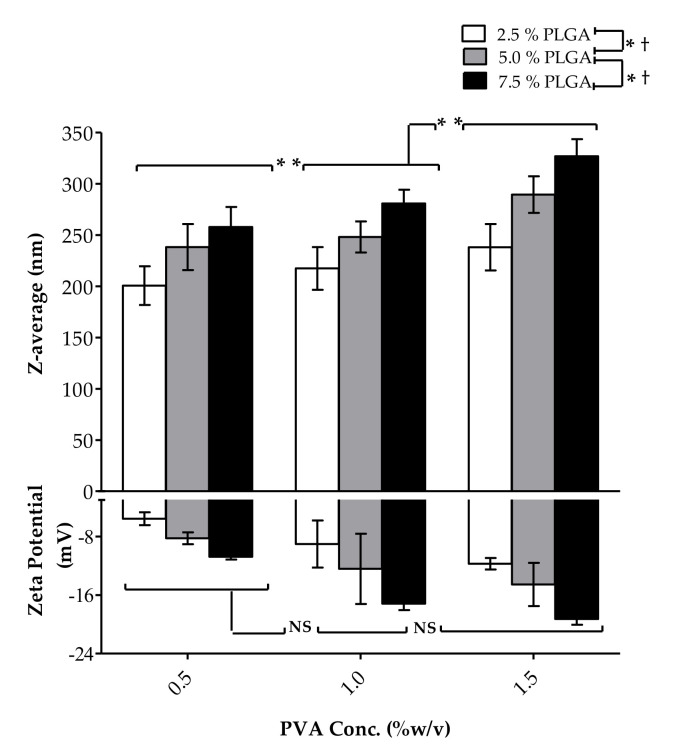
Effect of PLGA and PVA concentrations on the particle size and zeta potential of At-Ca NPs. Results show mean ±SD (n = 3). (* *p* < 0.05) and (** *p* < 0.05), the significant effect of increasing PLGA and PVA concentrations, respectively, on the particle size of At-Ca NPs. († *p* < 0.05) and NS, the significant and non-significant effects of increasing PLGA and PVA concentrations, respectively, on zeta potential value of At-Ca NPs.

**Figure 3:**
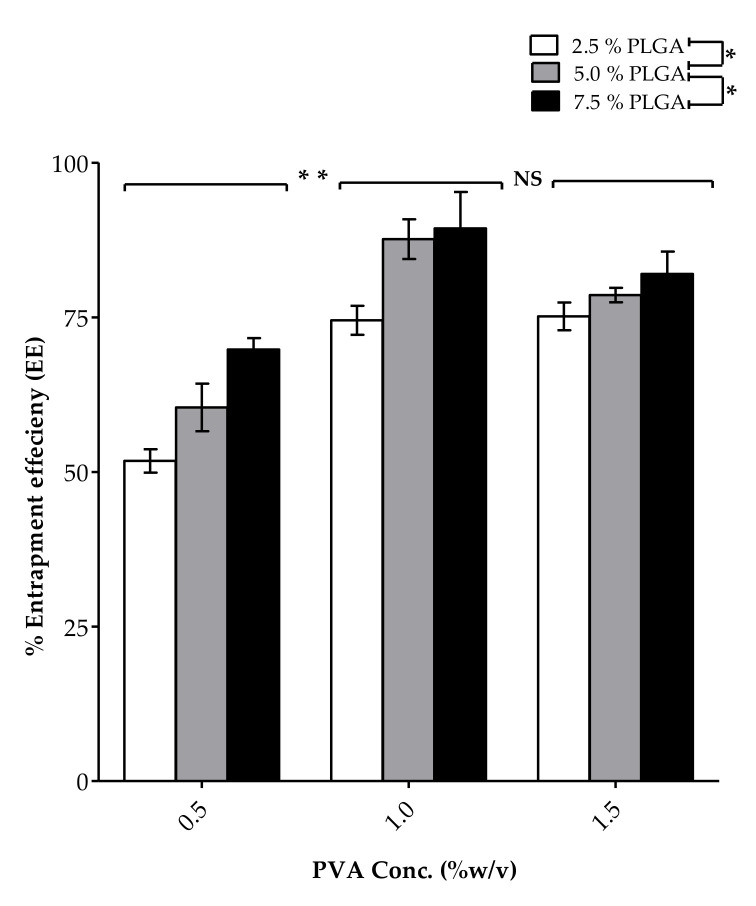
Effect of PLGA and PVA concentrations on the percent entrapment efficiency (% EE) of At-Ca NPs. Results show mean ±SD (n = 3). (* *p* < 0.05), the significant effect for increasing PLGA concentration. (** *p* < 0.05) and NS, the significant and non-significant effects for increasing PVA concentration to 1 and 1.5% *w*/*v*, respectively.

PLGA constitutes the main ingredient of the polymeric matrix encapsulating At-Ca. We utilized three different concentrations of PLGA at 2.5, 5 and 7.5% *w*/*v*. Increasing PLGA concentration significantly (*p* < 0.05) results in NPs with greater mean particle size (nm). As shown in Table 2 and Figure 2, the particle size of F7, F8 and F9 were equal to 238.1 ± 22.58, 289.5 ± 17.89 and 326.9 ± 16.74 nm for PLGA concentrations of 2.5, 5 and 7.5% *w*/*v*, respectively. In addition, variations in size distribution significantly increase with increasing PLGA concentration. PDI values for F1, F2 and F3 are equal to 0.096 ± 0.011, 0.134 ± 0.025 and 0.145 ± 0.012, respectively (Table 2), for the same previously mentioned different PLGA concentrations. Zeta potential of At-Ca NPs are highly controlled by PLGA concentration (Table 2 and Figure 2). Increasing PLGA concentration could significantly (*p* < 0.05) increase the net negative surface charge of the resulting NPs, for example F7, F8 and F9 have values of zeta potential equal to −11.71 ± 0.78, −14.55 ± 2.95 and −19.28 ± 0.77 mV, respectively. % EE significantly increases with increasing of PLGA concentration (Figure 3). Table 2 shows that % EE of F1, F2 and F3 is increasing by approximately 10% when PLGA concentration increases by 2.5% increments from 2.5% to 7.5% *w*/*v*.

#### 3.1.2. PVA Concentration

PVA is a common polymer widely used in multiple drug delivery systems due to its feasibility and biodegradability [24,25]. PVA was chosen as a surfactant added to the aqueous phase with varieties of concentrations (% *w*/*v*) as demonstrated in Table 1. Increasing PVA concentration from 0.5 to 1.5% *w*/*v* results in a significant (*p* < 0.05) increase in particle size (nm) (Table 2 and Figure 2). For example, the particle size of F6 and F9 are equal to 280.8 ± 13.55 and 326.9 ± 16.74 nm for PVA concentration of 1 and 1.5% *w*/*v*, respectively, at the same PLGA concentration equal to 7.5% *w*/*v*. NPs fabrication at higher PVA concentration have lower PDI with homogenous size distribution. As shown in Table 2, F2, F5 and F8 have PDI values equal to 0.134 ± 0.025, 0.126 ± 0.048 and 0.102 ± 0.029, respectively. The effect of PVA concentration on zeta potential of NPs are well illustrated in Table 2 and Figure 2 showing non-significant effect of PVA concentration on NPs net surface charge. PVA concentration has a diverse effect on % EE of NPs. A significant increase in % EE when PVA concentration increases from 0.5 to 1% *w*/*v*, whereas a non-significant effect observed on the values of % EE between PVA concentration of 1% compared to 1.5% *w*/*v* (Table 2, Figure 3). For example, % EE of F1, F4 and F7 were equal to 51.78 ± 1.89, 74.52 ± 2.35 and 75.15 ± 2.24 for PVA concentrations of 0.5, 1 and 1.5% *w*/*v*, respectively, with a significant difference (*p* < 0.05) between F1 and F4 and non-significant variation between F4 and F7.

### 3.2. Morphological Imaging

**Figure 4:**
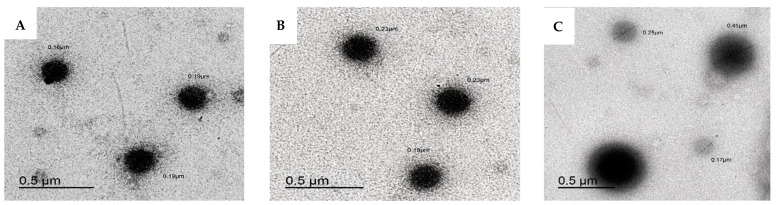
Transmission electron micrographs of At-Ca NPs. A, B and C for F1, F4 and F7, respectively. Formulae code is shown in Table 1.

Transmission electron microscopy (TEM) is utilized to visualize the geometry and shape of At-Ca NPs (Figure 4). NPs displayed spherical nanostructure with a clearly observed increase in particle size of F1, F4 and F7 equal to 180, 216.6 and 276.6 nm (the particle size is determined by calculating the mean of the individual particles labelled in Figure 4). These results are closely matched with the numerical values measured by laser diffraction technique (Table 2).

### 3.3. In Vitro Drug Stability

#### 3.3.1. Fourier-Transform Infrared Spectroscopy (FTIR)

**Figure 5:**
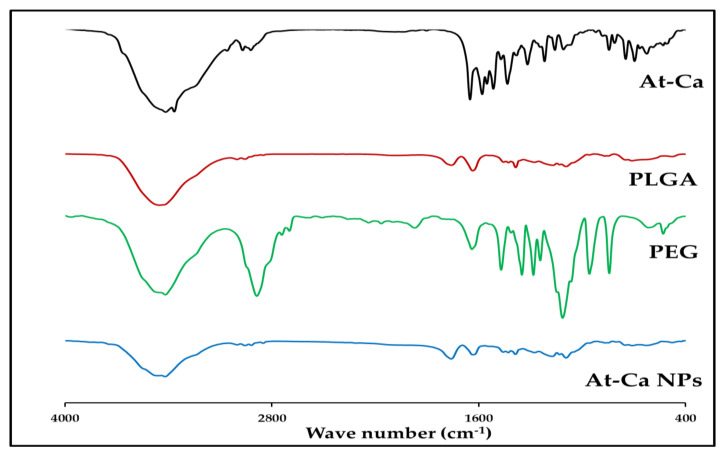
FTIR spectrum of free At-Ca, PLGA 50:50, PEG 2000 and At-Ca NPs (F5).

After fabrication of PLGA NPs encapsulating At-Ca, there is no major shifting in any of the characteristic peaks for all the components of PLGA, PEG and At-Ca. FTIR spectrum of At-Ca PLGA NPs (Figure 5) shows peaks at 3456 cm^−1^ (O-H stretching), 3001-2955-2850 cm^−1^ (C-H stretching), 1759 cm^−1^ (C=O stretching), 1457-1386-1275 cm^−1^ (C-H bending), 1173 cm^−1^ (=C-O asymmetric stretching ) and 1092 cm^−1^(C-O-C stretching ) that are closely matched with the positions of their corresponding peaks of PLGA spectrum (Figure 5, Table 3).

Furthermore, peaks at 3365 cm^−1^ (N-H stretching), 1634 cm^−1^ (C=O stretching), 1428 cm^−1^ (C-C stretching), 748 cm^−1^ (aromatic C-H out plane bending) and 627 cm^−1^ (C-H deformation bending) are presented on FTIR spectrum of At-Ca NPs (Figure 5) resembling the same characteristic peaks of free At-Ca (Table 3). It was demonstrated that peak at 3418 cm^−1^ become flatter and wider in At-Ca NPs spectrum indicating that hydrogen bond might be enhanced between O-H stretching peaks of PEG (3414 cm^−1^) and At-Ca drug (3416 cm^−1^) [34].

#### 3.3.2. Differential Scanning Colorimetry (DSC)

**Figure 6:**
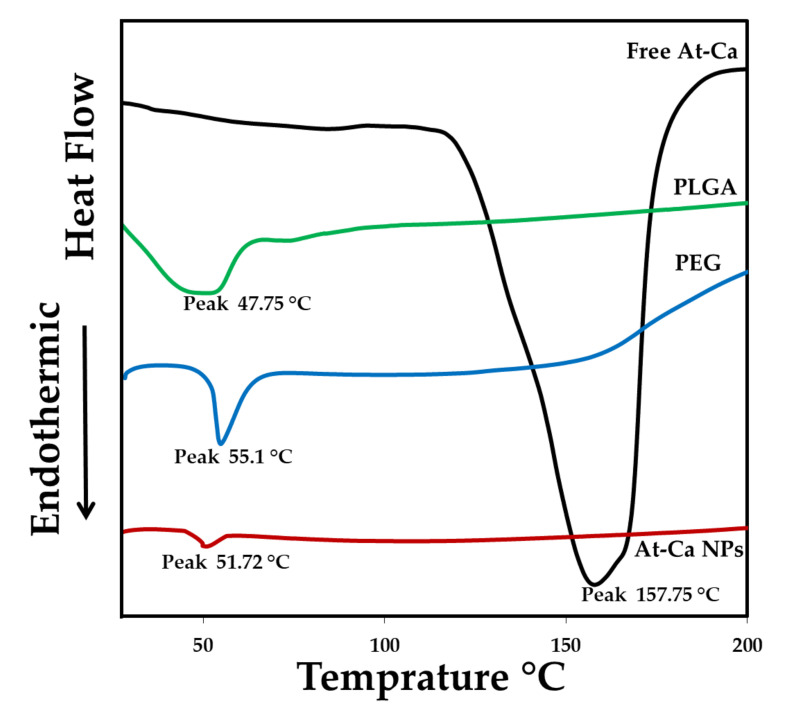
DSC thermograms for At-Ca, PLGA 50:50, PEG 2000 and At-Ca NPs (F5).

DSC study is mainly preformed to investigate the change in the physical state of At-Ca after encapsulation into polymeric nanocarriers. In our study free At-Ca, PLGA 50:50 and PEG 2000 show their endothermic peaks at 157.75 °C [7], 47.75 °C [35] and 55.1 °C [36,37], respectively (Figure 6). DSC thermogram of At-Ca NPs demonstrates an endothermic peak at 51.27 °C in a position similar to the individual peaks of PLGA and PEG with no peak in the range of 155 to 158 °C (Figure 6) indicating the disappearance of the characteristics At-Ca peak.

**Figure 7:**
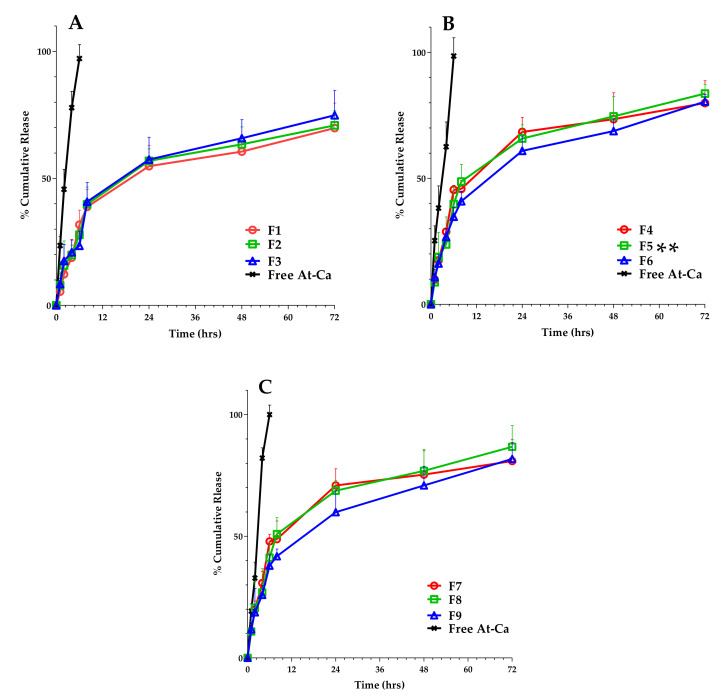
In vitro drug release profiles for At-Ca NPs (Formulae code shown in Table 1). For clarity, data are shown as mean + SD (n = 3). (**A**–**C**) patterns were for NPs formulations utilized PVA at concentrations of 0.5, 1 and 1.5 % *w*/*v*, respectively. At-Ca NPs (F5) has significantly higher initial burst and percent cumulative release (double asterisks, *p* < 0.05).

All At-Ca loaded NPs showed biphasic release platform with an initial burst at 6 h followed by sustained release profile extended to 72 h (Figure 7A–C). It is clearly observed that % cumulative release of free drug reached to approximately 100% at 6 h ensuring that dialysis membrane method offers no sustained effect for in vitro release study. All NPs formulations have significant (*p* < 0.05) more sustained release behavior compared with free At-Ca (control). At-Ca NPs with smaller diameter exhibits a significant higher initial burst, for example F7 (238.1 ± 22.58 nm), F8 (289.5 ± 17.89 nm) and F9 (326.9 ± 16.74) have initial bursts equal to 47.89 ± 2.89, 41.21 ± 5.71 and 37.89 ± 4.45%, respectively. Additionally, more sustained effect with greater percent cumulative release is significantly demonstrated for At-Ca NPs with higher values of % EE. As it is shown in Figure 7A,B, F1 (% EE = 51.78 ± 1.89) and F4 (% EE = 74.52 ± 2.35) display total percent cumulative release equal to 69.82 ± 9.78 and 79.89 ± 8.79%, respectively. Whereas their initial bursts are equal to 31.75 ± 5.76 and 45.46 ± 1.74 % for F1 and F4, respectively. This could be attributed to increasing PVA concentrations which significantly enhances higher initial burst values (Figure 7A–C). F5 was chosen an optimum formula for further in vivo study because it offers the highest percent cumulative release (83.63 ± 3.71) with high % EE and a well-controlled particles size concurrently with proper values of PDI and zeta potential (Table 2).

### 3.4. In Vivo Anti-Inflammatory Study

**Figure 8:**
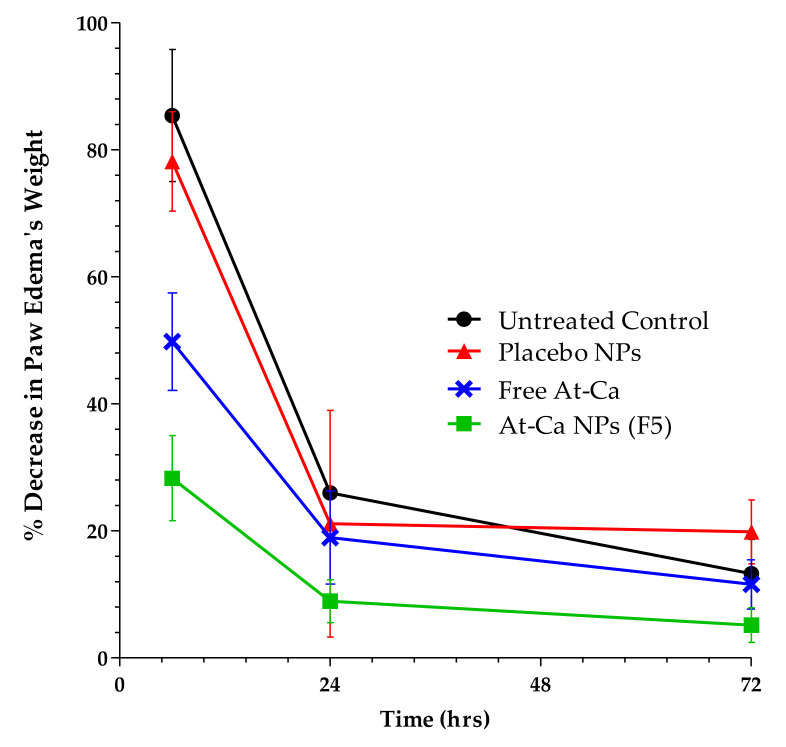
Percent decrease in paw edema’s weight after carrageenan induced inflammation of untreated control (circles), placebo NPs (triangles), free At-Ca (cross) and At-Ca NPs, F5 (squares) in male Sprague–Dawley rats. Results are expressed as means ± SD. (n = 6 per group).

The optimized At-Ca NPs (F5) showed a significant (*p* < 0.05) decrease in paw oedema in all time intervals compared to untreated control, placebo NPs and free At-Ca as demonstrated in Figure 8. The anti-inflammatory effect of both placebo NPs and untreated control is statistically non-significant producing similar results of percent decrease in edema’s weight. At 6 h time interval, it is obviously noted that free At-Ca has a significant reduction in edema’s weight compared with untreated control and placebo NPs with a non-significant difference in the remaining time intervals till 72 h (Figure 8).

**Figure 9:**
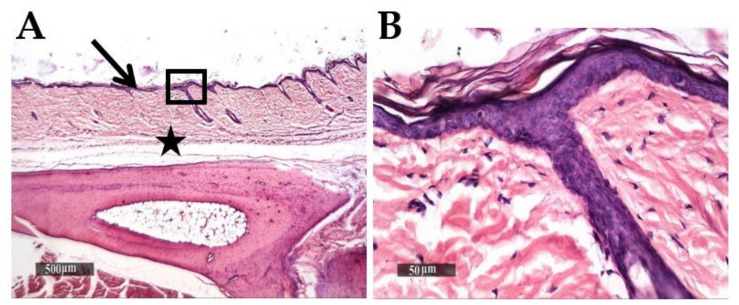
Normal morphological features of non-inflamed paws skin layers. H and E stain. (**A**) micrograph at ×40. Highlighted box in (**B**) micrograph at ×400. Black arrow and star show the epidermal layer and subcutaneous tissue, respectively.

Microscopic examination of normal non inflamed paws tissue demonstrated typical histological features of skin layers including intact epidermal layer consisting of apparent well-organized keratinocytes, intact dermal layer containing abundant collagen fibers and minimal inflammatory cells infiltrates with normal density of subcutaneous muscles and vasculatures (Figure 9A,B).

**Figure 10:**
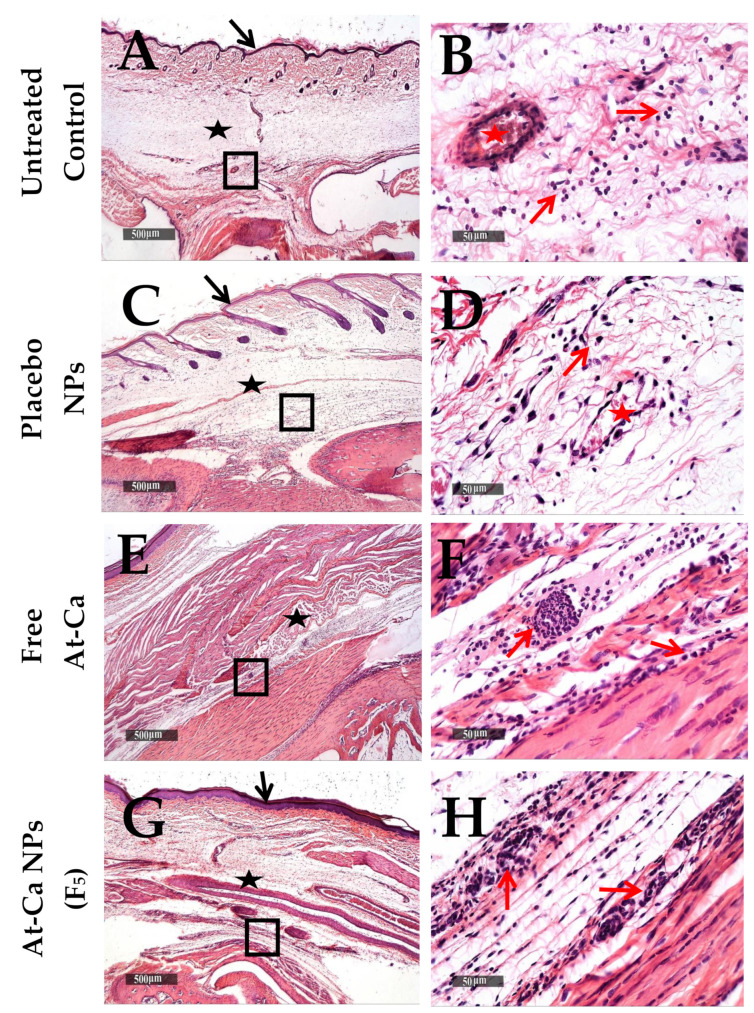
Histological changes of Paws skin layers in different groups (n = 6 per group), at 6 h time intervals after carrageenan injection. (**A**,**B**): untreated control, (**C**,**D**): Placebo NPs, (**E**,**F**): Free AT-Ca and (**G**,**H**): AT-Ca NPs (F5). H and E stain. (**A**,**C**,**E**,**G**) micrographs at ×40. Highlighted boxes (**B**,**D**,**F**,**H**) micrographs at ×400. Black arrows and stars show the epidermal layer and subcutaneous tissue, respectively. Red arrows and stars show inflammatory cells infiltrates and congested BVs, respectively.

**Figure 11:**
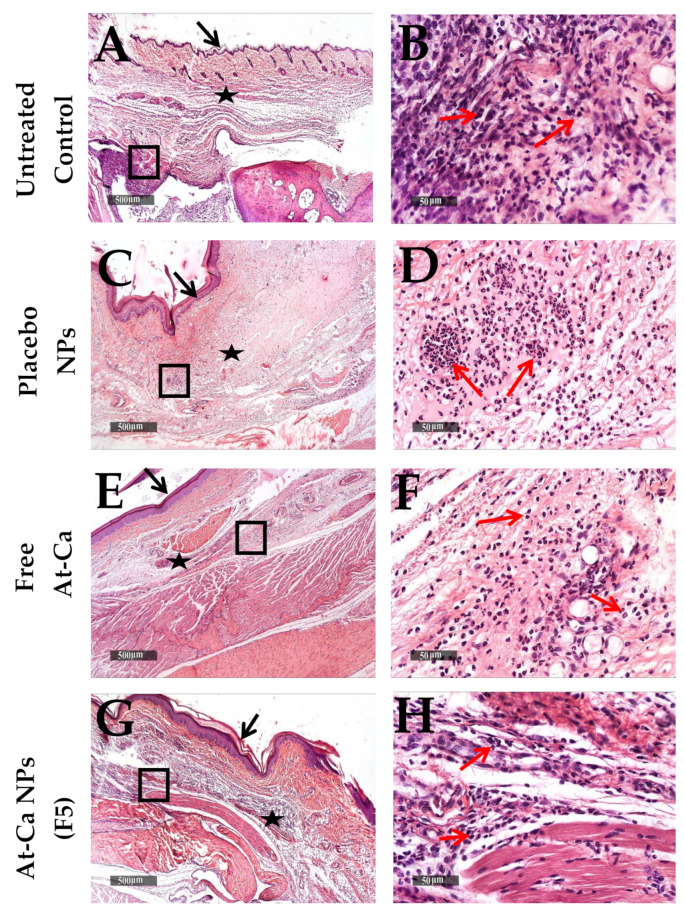
Histological changes of Paws skin layers in different groups (n = 6 per group), at 24 h time intervals after carrageenan injection. (**A**,**B**): untreated control, (**C**,**D**): Placebo NPs, (**E**,**F**): Free AT-Ca and (**G**,**H**): AT-Ca NPs (F5). H and E stain. (**A**,**C**,**E**,**G**) micrographs at ×40. Highlighted boxes (**B**,**D**,**F**,**H**) micrographs at ×400. Black arrows and stars show the epidermal layer and subcutaneous tissue, respectively. Red arrows show inflammatory cells infiltrates.

**Figure 12:**
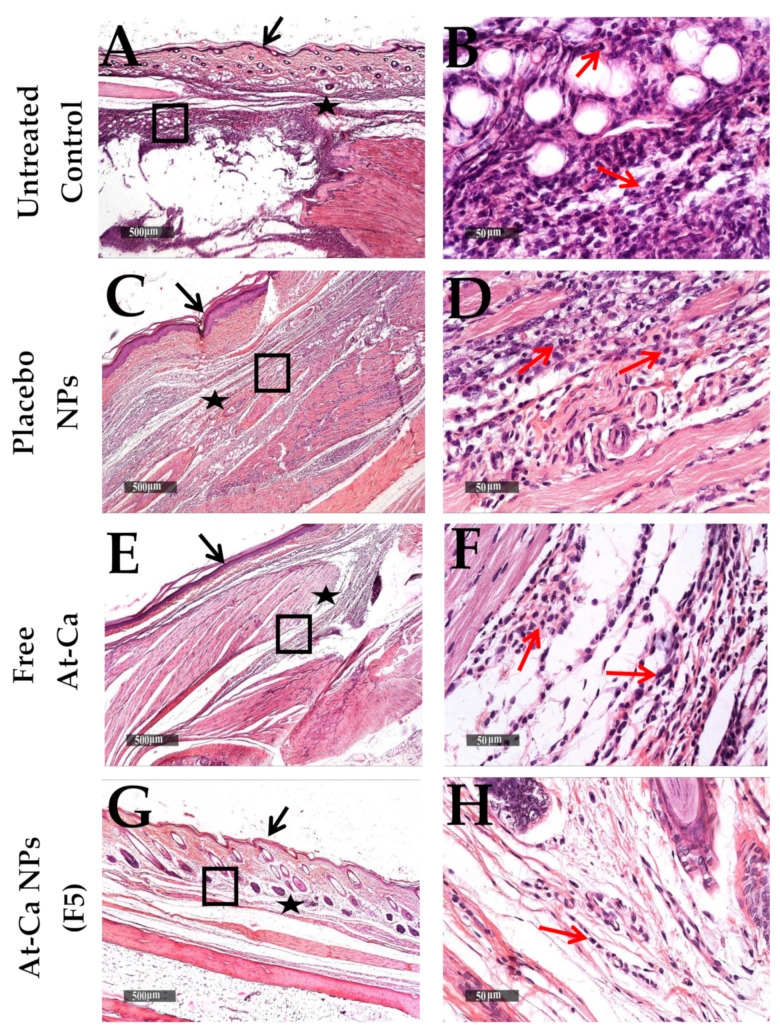
Histological changes of Paws skin layers in different groups (n = 6 per group), at 72 h time intervals after carrageenan injection. (**A**,**B**): untreated control, (**C**,**D**): Placebo NPs, (**E**,**F**): Free AT-Ca and (**G**,**H**): AT-Ca NPs (F5). H and E stain. (**A**,**C**,**E**,**G**) micrographs at ×40. Highlighted boxes (**B**,**D**,**F**,**H**) micrographs at ×400. Black arrows and stars show the epidermal layer and subcutaneous tissue, respectively. Red arrows show inflammatory cells infiltrates.

Sever subcutaneous and intermuscular edema, wide dispersion of dermal collagen fibers, numerous congested subcutaneous blood vessels (BVs) and mild to moderate increase of lymphocytic and neutrophilic infiltrates (Figure 10A–D) were demonstrated in both untreated control and placebo NPs tissue sections (Table 4A) at 6 h interval after carrageenan injection. Moreover, lower cellular infiltrates with less accelerated influx records were showed in deep subcutaneous tissue of free AT-Ca and AT-Ca NPs (F5) samples with milder severity of subcutaneous edema (Figure 10E–H and Table 4A). More sever diffuse subcutaneous inflammatory cells infiltrates and edema extends to periosseous connective tissue with many necrotic subcutaneous muscles were observed in control untreated samples (Figure 11A,B) at 24 h after carrageenan injection. Extensive subcutaneous edema and necrotic muscle fibers with several congested deep BVs and moderate records of neutrophilic inflammatory cells infiltrates were shown in tissue samples treated with placebo NPs (Figure 11C,D and Table 4B). However, Figure 11E–H showed more preserved morphological features with mild focal records of degenerated muscle fibers for free AT-Ca and AT-Ca NPs (F5). Significant milder (*p* < 0.05) subcutaneous edema was observed in AT-Ca NPs (F5) compared to a moderate record for free AT-Ca samples accompanied with moderate mixed inflammatory cells infiltrates (Table 4B). More pronounced subcutaneous persistence of inflammatory reaction was shown at 72 h compared with 24 h post injection interval in both untreated control and placebo NPs samples (Figure 12A–D) with some dissolution of subcutaneous edema in placebo NPs samples and minimal records of congested subcutaneous BVs (Table 4C). Non-significant reduction of subcutaneous edema (*p* > 0.05) was observed in free AT-Ca samples (Table 4B,C) accompanied with persistent level of mixed inflammatory cells infiltrates (Figure 12E,F) resembling 24 h samples. Significant protective efficacy (*p* < 0.05) of morphological structures with more accelerated normalization of subcutaneous tissue components and cellular elements were recorded in AT-Ca NPs (F5) samples with sporadic few records of inflammatory cells infiltrates, minimal subcutaneous edema and normal vasculature with no congested blood vessels were shown in more than 80% of examined samples (Figure 12G,H, Table 4C).

## 4. Discussion

PLGA utilized in At-Ca NPs fabrication is acid terminated functionalized with carboxylic acid groups. Generally, PLGA could be synthesized to be acid or ester terminated [38]. In our study we employed acid terminated PLGA because ester terminated PLGA could produce larger particle size and less stable NPs [39]. Acid terminated PLGA is more hydrophilic with negatively charged COOH and hence, more tendency to attract water molecules producing smaller particle size [39]. In addition, the interparticle electrostatic repulsion between similar negatively charged carboxylate groups might inhibit any possible aggregation between individually dispersed NPs leading to homogenous NPs distribution, better dispersibility parallel with increased stability [39]. PLGA concentration is a key controlling factor that significantly affects on particle size of At-Ca NPs. Higher concentration of PLGA increases the viscosity of the organic phase with greater oily droplet consistency which requires more shear force for proper emulsification. Coarser emulsion will be formulated because the homogenization power and stirring time are fixed at constant values throughout the study leading to NPs with bigger particle size [12]. Inadequate homogenization and poor drug dispersion occurred concurrently with increasing PLGA concentration which negatively affect on emulsification efficiency lead to non-uniformly particle size with high deviation in size distribution and PDI values [40]. The acidic contents of PLGA imparts a negative charge shielding NPs surface which is highly dependent on PLGA concentration, so increasing PLGA results in higher negative charge covering NPs surface offering greater electrostatic repulsion with better dispersibility [12,41]. % EE is an important in vitro parameter should be highly considered during evaluation of NPs because it mainly affects on release pattern of NPs and also, their in vivo implementation. Highly viscous organic phase obtained with increasing PLGA concentration leads to preventing drug molecules from diffusion out to the external surface with better incorporation into PLGA core which results in significant increase in % EE [12,42].

Increasing PVA concentration results in bigger NPs size. This could be attributed to formation of hydrophobic bonds between the hydrocarbon branches of PVA and the surface of PLGA NPs synchronously with spontaneous heavy inter/intra molecular hydrogen bonds constructed due to excessive hydration of PVA hydroxyl groups [12]. Higher concentration of PVA surfactant in the aqueous phase should enhance NPs hydrodynamic stability preventing their aggregation, therefore smaller deviation in size distribution and lower PDI values will be obtained [43]. Three washing cycles were carried out during NPs preparation to remove extra amount of PVA with micro levels might be attached on NPs surface which have negligible effect on zeta potential of NPs [12]. At lower PVA concentration equal to 0.5 and 1% *w*/*v*, PVA has a significant influence to increase % EE because the hydrophobic drug will be tightly encapsulated into PLGA internal core and its diffusion to the outer PVA surface will be reduced to the minimum. At higher PVA concentration of 1.5% *w*/*v*, the solubilizing power of PVA surfactant starts to improve drug solubility enhancing the drug leakage to the outer surface which leads to counteract drug desirability to be remained centralized in nanovesicle’s core [12,44].

PEG is nonionic polymer, universally employed during fabrication of NPs to enhance their residence time in blood circulation leading to achieve better absorption and hence enhanced pharmacokinetic properties of nano formulations [45]. There are different methods used to incorporate PEG into polymeric PLGA NPs. Chemically synthesized coblock polymers is the main approach [45] and also, physically comixing PLGA with PEG is considered a promising strategy which efficiently affects on NPs physicochemical features [12,19]. Here in this study, we added comixed PEG for surface modification because PEG could form a periphery layer surrounding NPs. This layer acts as biological barrier protecting the nano system from macrophage engulfment which leads to higher stability and delayed degradation [19]. PEG plays a crucial role in improving in vitro characterization of NPs. Association of polymeric networks modified after PEG addition might produce NPs with smaller diameter [12,19,46]. As our model drug At-Ca is highly hydrophobic, its entrapment will efficiently increase into PLGA hydrophobic core with no tendency for diffusion out to the external hydrophilic PEG surface [47]. Additionally, PEG controls the prolonged release of At-Ca NPs for 72 h due to reducing drug release rates and slowing down its diffusion outside the nanovesicle [47]. PEG aids to exhibit sustained anti-inflammatory action of At-Ca PLGA NPs.

FTIR is as important analytical study should be fully investigated during NPs preparation to exclude any physical and chemical interaction might occur between the polymeric material and drug, also, to ensure the compatibility between all the components of NPs. FTIR aids to examine the effectiveness of drug encapsulation into polymeric core of NPs [7,45]. As illustrated in Section 3.3.1, FTIR spectrum of AT-Ca NPs shows no loss of the functional groups of free drug and native PLGA negating any chemical interaction occurred between the drug and polymer. This results proves that our fabrication method used for NPs preparation is a successful technique protecting the drug stability and integrity [7]. Most of detection peaks regarding to PEG spectrum were not clearly observed on At-Ca NPs spectrum. The spectral bands of PLGA (major component of polymeric matrix) might mask the spectral peaks of PEG existed in trace concentration comixed with PLGA [48]. DSC thermogram of At-Ca NPs elucidates that there is no drug in crystalline form after encapsulation into PLGA core. This ensures that At-Ca might be in amorphous form or dispersed homogeneously in polymeric matrix [7,34]. Additionally, this results demonstrate that At-Ca was efficiently dissolved in the organic phase during NPs preparation with no tendency to precipitate after solvent evaporation due to the polymeric matrix sufficiently surrounding drug molecules and preventing their aggregation and/or crystallization [7].

Biphasic release pattern of PLGA NPs could be explained by the initial burst firstly emerged due to the presence of loosely bound drug molecules on the outer surface that is highly enhanced by efficient wettability offered by PVA [49]. The second sustained release phase manifested by PLGA polymer showed a delayed release extended for three days [12,49]. In vitro release At-Ca from PLGA NPs is highly dependent on particle size. Smaller particles with larger surface area exposed to release media facilitate drug diffusion which results in higher initial bursts values [7,12]. Even though At-Ca PLGA NPs were orally administrated, pH of the release medium was adjusted to 7.4. Intact NPs would be absorbed directly into blood circulation (pH 7.4) via payer patches’ pathway. All in vitro release experiments were performed in pH 7.4 simulating the in vivo condition [50].

The results of in vivo study revealed the sustained anti-inflammatory effect of At-Ca PLGA NPs [19,51] extended for approximately three days compared with free At-Ca which has rapid onset of action lasted for only six hours. Histological examination showed that the paw skin tissue could restore its normal histological structure of epidermal/dermal layers after oral administration of AT-Ca NPs within 72 h. Placebo NPs have similar results of untreated control which negates any anti-inflammatory effect for PLGA and PEG forming the main matrix of the polymeric nanoarchitecture.

## 5. Conclusions

The anti-inflammatory effect of At-Ca is considered a novel pharmacological action that could be widely employed in the near future. Polymeric PLGA NPs loaded with At-Ca provide excellent in vitro features offering a sustained release platform for 72 h. Normal density of subcutaneous muscles, minimal inflammatory cells, healthy blood vessels and no subcutaneous oedema were clearly observed in Paw’ tissues of male Sprague–Dawley rats after orally administration of At-Ca PLGA NPs comparing with placebo NPs (contain no medication) and free drug.

## Figures and Tables

**Table 1:** Formulae coding and composition of At-Ca PLGA NPs.

Formula Code	PLGA Concentration (% *w*/*v*)	PEG Content in Primary Emulsion (% *w*/*w*)	PVA Concentration in Aqueous External Phase (% *w*/*v*)	Drug Loading (mg)	Organic/Aqueous Phase Volume Ratio
F1	2.50				
F2	5.00	5.00	0.50	10	1:100
F3	7.50				
F4 F5 F6	2.50 5.00 7.50	5.00	1.00	10	1:100
F7 F8 F9	2.50 5.00 7.50	5.00	1.50	10	1:100

**Table 2:** Physicochemical properties of At-Ca PLGA NPs formulations (F1–F9). Data represent mean values ± SD of at least three measurements.

Formula Code	Z-Average	Polydispersity Index (PDI)	Zeta Potential (mV)	% EE
	(nm)			
F1	200.7 ± 18.85	0.096 ± 0.011	−5.57 ± 0.88	51.78 ± 1.89
F2	238.3 ± 22.43	0.134 ± 0.025	−8.23 ± 0.82	60.42 ± 3.84
F3	257.9 ± 19.53	0.145 ± 0.012	−10.77 ± 0.36	69.81 ± 1.84
F4	217.5 ± 20.87	0.107 ± 0.066	−9.02 ± 3.22	74.52 ± 2.35
F5	248.2 ± 15.13	0.126 ± 0.048	−12.41 ± 4.80	87.63 ± 3.21
F6	280.8 ± 13.55	0.151 ± 0.039	−17.18 ± 0.87	89.36 ± 5.89
F7	238.1 ± 22.58	0.076 ± 0.047	−11.71 ± 0.78	75.15 ± 2.24
F8	289.5 ± 17.89	0.102 ± 0.029	−14.55 ± 2.95	78.95 ± 1.17
F9	326.9 ± 16.74	0.110 ± 0.012	−19.28 ± 0.77	82.02 ± 3.59

**Table 3:** Characteristic IR peaks of PLGA 50:50, PEG 2000 and At-Ca.

	Assignment	Approximate Frequency (cm^−1^)	Description
PLGA 50:50 [26,27,28]	O-H (very broad)	3452.21	Stretching
	C-O-C	1093.27	
	=C-O	1169.23	Asymmetric Stretching
	C-H	3001.64 (CH3)	Stretching
		2955.70 (CH2)	
		2851.21 (CH2)	
		1455.76 (CH3)	Bending
		1385.24 (CH3)	
		1276.49 (CH2)	
	C=O	1758.52	Stretching
		1427.20 1385.24	Bending
PEG [29,30,31]	O-H (very broad)	3414.43	Stretching
	C-H	2886.97	
	C-O	1112.8	
	C-C	842.13	
At-Ca [7,32,33]	O-H (very broad)	3416.25	Stretching
	N-H	3366.44	
	C-H	3059.59 2969.41 29922.27	
	C=O	1649.89 1579.24	
	C-C	1515.04 1433.1	
	CH3 and -CH2	1316.51	Deformation Bending
	C-N	1217.72	Stretching
	C-O	1157.55	
	aromatic C-H	843.52 747.00	Out plane Bending
	C-H	695.58 623.99	Deformation Bending

**Table 4:** Score records of histological examined lesions after carrageenan injection at different time intervals. A, B and C for 6, 24 and 72 h, respectively.

A Inflammatory cells infiltrates Subcutaneous oedema Congested BVs	**Untreated Control**	**Placebo NPs**	**Free AT-Ca**	**AT-Ca NPs (F5)**
	+++ +++ ++	++ +++ ++	++ ++ ++	++ ++ ++
B Inflammatory cells infiltrates Subcutaneous oedema Congested BVs	**Untreated Control**	**Placebo NPs**	**Free AT-Ca**	**AT-Ca NPs (F5)**
	+++ +++ ++	++ +++ ++	++ ++ +	++ + +
C Inflammatory cells infiltrates Subcutaneous oedema Congested BVs	**Untreated Control**	**Placebo NPs**	**Free AT-Ca**	**AT-Ca NPs (F5)**
	+++	+++	++	+
	++	++	++	+
	++	+	+	-

- Nil (no lesions were demonstrated). + Mild lesion recorded in less than 15% of examined tissue sections. ++ Moderate lesion recorded in 16–35% of examined tissue sections. +++ Sever lesion recorded in more than 35% of examined tissue sections.

## Data Availability

All the data of this research are available upon request.
